# IoMT Platform for Pervasive Healthcare Data Aggregation, Processing, and Sharing Based on OneM2M and OpenEHR

**DOI:** 10.3390/s19194283

**Published:** 2019-10-03

**Authors:** Jesús N. S. Rubí, Paulo R. L. Gondim

**Affiliations:** Department of Electrical Engineering, University of Brasilia, Brasilia 70910-900, Brazil; nsuarezrubi@aluno.unb.br

**Keywords:** e-Health, pervasive healthcare, OpenEHR, M2M, IoMT, semantic interoperability, data warehouse, data mining, FHIR

## Abstract

Pervasive healthcare services have undergone a great evolution in recent years. The technological development of communication networks, including the Internet, sensor networks, and M2M (Machine-to-Machine) have given rise to new architectures, applications, and standards related to addressing almost all current e-health challenges. Among the standards, the importance of OpenEHR has been recognized, since it enables the separation of medical semantics from data representation of electronic health records. However, it does not meet the requirements related to interoperability of e-health devices in M2M networks, or in the Internet of Things (IoT) scenarios. Moreover, the lack of interoperability hampers the application of new data-processing techniques, such as data mining and online analytical processing, due to the heterogeneity of the data and the sources. This article proposes an Internet of Medical Things (IoMT) platform for pervasive healthcare that ensures interoperability, quality of the detection process, and scalability in an M2M-based architecture, and provides functionalities for the processing of high volumes of data, knowledge extraction, and common healthcare services. The platform uses the semantics described in OpenEHR for both data quality evaluation and standardization of healthcare data stored by the association of IoMT devices and observations defined in OpenEHR. Moreover, it enables the application of big data techniques and online analytic processing (OLAP) through Hadoop Map/Reduce and content-sharing through fast healthcare interoperability resource (FHIR) application programming interfaces (APIs).

## 1. Introduction

The aging and growth of the world’s population has caused traditional health information systems to become more complicated. Today, addressing the spread of the modern healthcare information systems to cover peripheral areas, while making accurate predictions of major diseases, are critical challenges. The rapid development of mobile computing, wireless communication, sensor networks, and embedded systems has also resulted in health information systems approaching the era of pervasive computing. This has led to a constant growth of applications and devices (i.e., wearable devices) for providing new pervasive health services [1], which are rapidly being adopted worldwide [2].

On the other hand, the Internet of Things (IoT) [3] provides computer power and Internet connections to everyday objects, capable of processing, communicating, and storing the data captured from the physical world. Most of the applications of IoT are pervasive in nature [4]. More specifically, in the healthcare domain, IoT applications have led to the concept of the Internet of Medical Things (IoMT) [5] (also known as healthcare IoT). This refers to a global infrastructure composed of medical devices and applications interconnected through the Internet. In this context, sensors for physiological parameters (i.e., ECG, blood pressure, blood oxygen saturation, etc.), behavior (i.e., pedometers, gyroscopes, GPS, etc.) and patient context (i.e., relative air humidity, temperature, etc.) enable continuous observation and monitoring of health-related parameters. As a consequence, healthcare professionals can offer more accurate and lower-cost services without intervening in the patients’ daily routine.

IoMT-based platforms can empower the patient in the sense that they become self-aware of their health status [6]. In many cases, they even avoid or minimize the intervention of healthcare professionals. For example, Verma and Sood [7] proposed a cloud-centric IoT-based framework for monitoring disease diagnosing which automatically predicts potential diseases and their level of severity without healthcare professionals participating. Moreover, these platforms empower the physicians with technologies that simplify the healthcare process in addition to providing them with tools to support clinical decisions. This has made IoMT widely used to enhance healthcare and is now considered to be a pillar in new pervasive health services [8].

However, the number of IoMT devices is expected to grow considerably in the coming years and the heterogeneity present in different IoMT components (network interfaces, communication protocols, data structure, data semantics) will impose interoperability and privacy related challenges [9]. In this sense, a pervasive healthcare platform must be flexible enough to handle all these concepts. Therefore, the integration of IoMT solutions in an interoperable environment and the development of tools for the storage, processing, and widespread dissemination of IoMT data have become relevant. They have also required overcoming technological barriers that hamper the design of new solutions. The following open issues are related to the heterogeneity of devices and possible data representations, as well as the volume and complexity of the collected data:

1. Standardization in the context of pervasive healthcare still does not guarantee interoperability among IoMT solutions [10], which is a general problem in IoT. Each manufacturer uses its own definitions of devices, data format, and communication protocols, which hampers integration with larger systems. Moreover, many solutions are closed and do not implement functionalities for data interchange;
2. Several communication protocols have been used in IoT/IoMT and their performances depend on the functionalities they offer. Depending on the application context, one protocol may be more suitable than another (i.e., a publish/subscribe-based for monitoring or a client/server-based protocol for data aggregation). An IoMT platform must be adaptable in such a way that enable the use of existing communication protocols and the integration of new ones [11]. However, there is a lack of proposals that faced this issue and there is a tendency for the definition of platforms that considers only one communication approach (not standardized in many cases);
3. Electronic health records [12] (EHRs) have been widely used for storing patient data in hospital information systems. However, the lack of proposals for their integration with IoMT platforms [13] has caused health professionals to manage a patient’s clinical history with distributed records provided by multiple sources. This requires the knowledge of several platforms and applications, in addition to human effort and time for migrating information and data among them; and
4. The volume and complexity of the data collected in IoMT solutions hamper their interpretation by physicians, hence, leading to difficulties in dealing with big data. Such challenges advocate the use of intelligent algorithms that correlate vital signs and extract valuable insights hidden in healthcare data repositories [14]. Even though big data techniques, such as Map/Reduce [15], linear regression, classification, and clustering have become popular solutions, the lack of standards for data integration and standardization impede their application.

Several studies have aimed at solving such issues. Rajkomar et al. [16] analyzed the impact of health data source heterogeneity on the application of data processing and knowledge-extraction techniques (i.e., training of machine learning models for decision support). The representation of data collected for granting compatibility among different IoMT platforms and their sharing among different applications is highly relevant.

An EHR maintains a patient’s medical history using a unique identifier, e.g., the national health ID, for relating a patient’s health data. It can be implemented locally, within a single institution, or developed in more complex structures (i.e., at country or international level). Its main objective is to facilitate access to information in relation to a wide range of new applications and architectures that follow the modeling of the data adopted in the EHR.

Organizations, such as OpenEHR [17], have standardized EHRs models. However, their use involves collection, transformation, and loading of data handled by heterogeneous IoMT solutions. This process can be conceptualized as “Collect-Transform-Load” process (CTL). Nevertheless, it must be studied, since each manufacturer uses proprietary protocols, which hinders their integration with larger systems. Therefore, a CTL mechanism standardized among sensors, IoMT devices and EHRs is required.

An IoMT platform must also provide mechanisms for data sharing with third party applications. Standards, such as those developed by Health Level 7 [18] (HL7), enable data sharing among various health systems and applications. However, the EHR data must be translated to HL7-compatible resources.

Applications focused on knowledge extraction that work with a large volume of data require more than an API, in order to prevent latency. The platforms must provide mechanisms for the application of data mining and data warehousing techniques that help physicians through clinical decision support systems (DSS).

In summary, a modern pervasive healthcare platform should provide, at the minimum, functionalities for:

1. Storage and management of clinical and demographic data in a standardized EHR in relation to their organization in a human-understandable approach;
2. A CTL mechanism for the integration of multiple IoMT solutions which consider heterogeneous communication protocols;
3. Management of patient clinical data by medical teams (creation, visualization, update and deletion of electronic records); and
4. Knowledge extraction and analytical studies to support clinical DSS services (for example, by the use of OLAP and-or data mining and-or data warehousing techniques).

Additionally, isolated Personal Healthcare Devices (PHDs) that collect valuable healthcare data, such as fitness bands, pedometers, heart rate sensors in smart watches, among others, require a functional architecture for data collection. Standards, such as M2M communications developed by the European Telecommunication Standards Institute [19] and its extension on the OneM2M standard [20] defined the main functionalities of this type of architecture.

Computer paradigms, as cloud computing, data warehousing, big data and data mining, in addition to standards, such as OpenEHR and OneM2M, enable the definition of an architecture and a platform that meet the requirements listed above.

Taking the aforementioned issues into consideration, this article introduces a platform that which: favors the creation of new IoMT applications; grants interoperability from the collection to the publication of data; integrates multiple communication protocols; provides functionalities for data warehouse and application of OLAP techniques; and provides tools for data mining through Hadoop. It is based on an extension of OpenEHR semantics for the IoMT domain, following an OneM2M communication architecture which integrates IoMT devices to an OpenEHR-based EHR. Moreover, a performance study regarding the communication protocols considered by the platform is provided.

The main contributions of the platform include:

1. Extension and integration of OpenEHR semantics to the IoMT domain for simplifying the collection and dissemination of data and enhancing IoMT interoperability;
2. CTL programming tools for IoMT solutions and data from isolated devices;
3. Simplified data inspection using OLAP features; and
4. Simplified healthcare knowledge extraction through data mining and machine learning capabilities.

A development framework is also provided for the integration of sensors and healthcare systems to the platform with little effort. It is extensible in terms of sensors and communication protocols. Furthermore, its abstraction layer can be extended to meeting the specific requirements of these components. The framework has already implemented the Message Queuing Telemetry Transport (MQTT), Constrained Application (CoAP) and Representational State Transfer over HTTP (REST HTTP) communication protocols and a performance analysis is provided.

As for the structure of the article, it is organized as follows: Section 2 addresses related works and introduces the basics for the platform definition; Section 3 describes the proposed platform and evaluates its performance; finally, Section 4 provides the conclusions and proposes future studies.

## 2. Related Works

As addressed elsewhere, interoperability in pervasive healthcare impacts directly on different levels, such as (i) interoperability of the collection system, involving sensor networks and integration of heterogeneous IoMT devices; (ii) interoperability in the exchange of data stored in heterogeneous IoMT information systems; (iii) definition of mechanisms for the dissemination of data to third party applications; and (iv) services for massive data processing and knowledge extraction. Below are some studies that partially solve such issues, however without considering the whole scenario.

Boutros-Saikali et al. [21] developed a platform that integrates different IoMT solutions for companies, such as Samsung, Microsoft, and Nokia. Data are collected by connectors, which are specific programs for a systematic extraction of data from each provider. The data are then changed to a format not specified in the article, stored in a non-standardized EHR, and re-transformed to resources in FHIR (fast healthcare interoperability resource) [22] and Open mHealth (author-defined format). The FHIR resources can be queried by third parties through a set of REST APIs defined by the FHIR standard (see Section 2.2). However, the platform does not consider the integration of isolated IoMT devices and ignores the supply of an environment that enables the application of data mining or analytic processing.

Other proposals enable an agent-based approach to integrate isolated IoMT solutions. An agent plays the same role of the connector in the previous approach, which performs the CTL process, but follows an Agent-oriented programming (AOP) paradigm [23].

Cardoso et al. [24] designed AIDA platform, in which an agent is developed to interact with each type of medical equipment and forwards the collected data to a main server. The authors used JADE framework [23] and, apparently, the EHRs are based on FHIR. However, the study lacks a definition of tools for data processing and integration of other EHRs, rather than IoMT devices.

Kim [25] defined an agent-based platform in which a set of agents (monitoring, component and system agents) performs the CTL from primitive platforms. Another set of agents (diagnosis, decision and searching agents) queries data and performs operations for clinical DDS. Data do not remain on the platform and are retrieved by the agents when necessary. The process hampers the application of data-processing techniques, since the massive volume of data must be retrieved in real time.

Guan et al. [26] developed Vicinity platform, which provides interoperability as a service following the concepts of adapters. Adapters can be consumed as services on demand and act similarly to the connectors defined in [21]. The main difference is connectors are developed specifically for a set of communication and data format standards, which simplifies the integration of IoMT devices to the platform. However, it hampers integration with IoMT systems that do not follow a standardized approach.

Irfan and Ahmad [27] conducted a survey on IoMT architectures and observed that two to five layers were commonly used. The authors adopted a three-layered IoMT architecture composed of things, intermediate, and integrated application layers to cover all IoMT requirements. The Things layer is comprised of heterogeneous IoMT devices that communicate through various communication protocols and networks. The Intermediate layer is represented by a middleware or gateway that handles the IoMT devices and processes the data at a local stage. The Integrated Application layer stores huge amounts of data and processes them through several applications. The authors ignored the definition of EHRs and disregarded the volume of data involved in the IoMT scenario.

Those studies help identify a base architecture for IoMT integration where data are collected by heterogeneous IoMT devices and forwarded to gateways and local servers near an IoMT solution (at the network edge) or adapters, connectors or agents (instantiated either at the network edge, or in the cloud), which transform the heterogeneous data formats into a unified one. The collected data are then loaded to the cloud through services or in place when the adapters, connectors, or agents are instantiated in the cloud. Finally, the external applications access the data through application services, or according to an agent-based approach.

The architecture shows several shortcomings regarding the functional requirements previously defined, particularly those related to massive data processing and data standardization that uses an EHR definition.

On the other hand, Pramanik et al. [28] proposed a big data-based smart pervasive healthcare system framework that offers knowledge-extraction capabilities over a data repository. Differently from the other approaches, it does not consider data sharing for third party applications and a standardized EHR.

**Figure 1:**
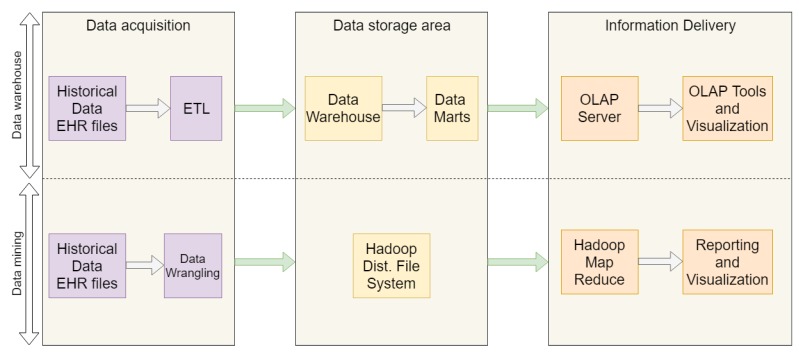
Data warehousing, OLAP and big data approaches (adapted from [29]).

Essa et al. [29] analyzed the use of data-processing platforms in multiple OpenEHR-based EHRs following two approaches (Figure 1) that consider three layers, namely data acquisition, data storage, and information delivery. The first considers the Extract-Transform-Load (ETL) process from multiple EHR files into a data warehouse that serves an online analytical processing (OLAP) [30] server which provides analyses and visualization tools.

The second approach enables the application of data mining techniques through Hadoop Map Reduce [15] supported by data stored in a Hadoop distributed file system (HDFS), populated with EHRs records after a data wrangling process. As with ETL, data wrangling (DW) [31] applies data profiling, cleaning, transformation, integration and visualization operations to improve the quality of data, and corrupted, repeated or incoherent EHR records are filtered towards better quality data for data mining applications.

However, the platforms are closed and do not provide functionalities for data interchange with external applications, which incurs in the above-defined prerequisites for IoMT.

Table 1 shows a summary of the related works addressed in this section. No approach could cover the functionalities elicited for IoMT, which together with the concepts introduced below have motivated us to propose a new platform for IoMT.

### 2.1. OneM2M Communications

The main objective of OneM2M [32] is to develop technical specifications for a common M2M service layer, thus minimizing standards market fragmentation. It has consolidated the isolated M2M service layer standards and developed a global specification. The benefits offered by OneM2M to the M2M ecosystem include shortened time to market, since common components do not need to be developed, which simplifies the creation of applications, and a common set of application programming interfaces (APIs).

**Figure 2:**
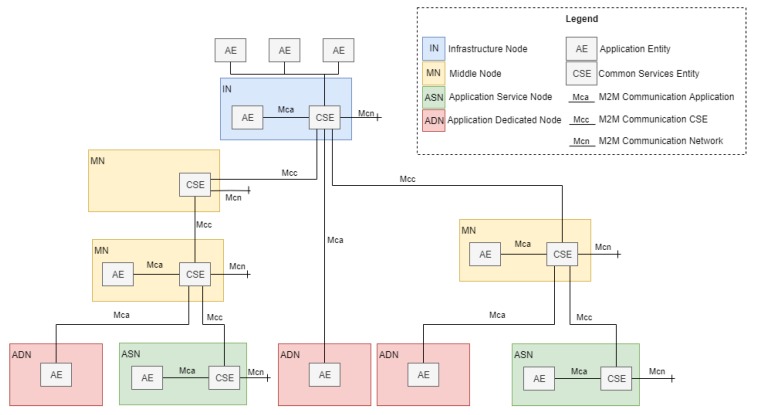
OneM2M functional architecture.

Figure 2 shows the functional architecture formed by functional entities called nodes: application dedicated node (ADN), application service node (ASN), middle node (MN), and infrastructure node (IN). Nodes host at least one OneM2M common services entity (CSE) or one OneM2M application entity (AE) [32]. A CSE is a logical entity instantiated in an M2M node and comprises a set of service functions called common services functions (CSFs). CSFs can be used by applications and other CSEs. An AE is a logical entity that provides application logic, e.g., remote blood sugar monitoring, for end-to-end M2M solutions.

OneM2M currently defines three reference points (Mca, Mcc, and Mcn), where the “Mc” prefix stands for M2M communication. The Mca reference point enables AEs to use the services provided by the CSE, and the Mcc reference point enables inter-CSE communications [32]. The Mcn reference point is located between a CSE and the service entities in the underlying networks, such as the device triggering service provided by Third Generation Partnership Project (3GPP) networks.

OneM2M does not restrict the Mca, Mcc, and Mcn underlying communication protocols. The “Binding Protocol” concept, which represents application and transport layer protocols, such as MQTT, CoAP, or REST over Http, refers to the communication between nodes. Working Group WG3 is a developing standardization of bindings for flows for specific transport protocols [33], as those previously addressed.

By separating the service layer from the communication layer, OneM2M favors the platform’s adaptability and integration with heterogeneous devices and network configurations. This is a key aspect considering the constant evolution of communication protocols for IoMT and the efforts required for its integration with the platform.

Another important characteristic of OneM2M is its origin. OneM2M is based on the analysis of a set of use cases [34] that involve M2M communications and encompass all their requirements. Particularly for healthcare, three use cases taken considered only integration between devices and applications and disregarded integration with hospital information systems. Moreover, the infrastructure node did not consider tools for data processing. In this sense, we decided to follow a OneM2M-based functional architecture for IoMT device integration, but extended it to fulfill the requirements of the proposed platform.

In terms of infrastructure and technologies for implementation of M2M, Kartsakli et al. [35] made an extensive review of wireless communication technologies used in M2M-based healthcare solutions. Some of these technologies were considered for the construction of our testbed, as Wi-Fi and LTE.

### 2.2. FHIR

FHIR enables the exchange of information for the delivery of healthcare in a wide variety of settings. The specification is based on Web resources and adapts modern and widely used RESTful practices for the delivery of integrated medical care across a broad range of teams and organizations.

The intended scope of FHIR covers human and veterinary aspects, clinical care, public health, clinical trials, administration, and financial aspects for a global use in several architectures and scenarios. It is based on “Resources”, which are the common foundations for all exchange and an instance-level representation of some type of healthcare entity. Their common features include a URL that identifies the resource common metadata, a human readable XHTML summary, a set of defined data elements, a different set for each type of resource, and an extensibility framework that supports variations in healthcare. Resource instances are represented as XML, JSON, or RDF, and total 145 different types defined in the FHIR specification, which describes those that can be exchanged. The term “Resource” is sometimes used with no clarification on whether it refers specifically to types or instances; however, the context clarifies it.

### 2.3. OpenEHR

OpenEHR [17] is an open standard intended for electronic health record systems. Based on a multi-level modeling approach, it separates medical semantics from data representation. This is done so that domain experts can be directly involved in the semantics and define models known as archetypes and processes of clinical information systems using a much easier terminology.

Its structure can be defined according to a three level/layer approach. The reference model is the base layer and contains definitions of data structures, types, security aspects, and an identifying attribute that supports the indexation of each reference model. The next layer contains the models related to medical semantics, as archetypes (defined in a computer language, called ADL (Archetype Definition Language)) and templates. The top layer provides different services to the external context according to a service-oriented architecture.

Below is a brief discussion on archetypes and templates for a better understanding of the OpenEHR structure.

Different archetypes can be grouped into a template aimed at more complex definitions. This means that an archetype may refer only to a measure of a single physiological parameter, whereas a template can refer to a complete physiological parameters screening of a cardiovascular disease which includes archetypes for blood pressure, heart rate, electrocardiographic signals, etc. A template is often used for the definition of a case report form or a form to be filled. It enables the determination of the constructs to be used, events that must exist for each construct, elements to be used within an archetype, among other settings.

Another interesting characteristic is the existence of global repositories that provide standardized archetypes in different languages. This is an advantage in terms of interoperability and re-use of source code and applications. Moreover, this centralized distribution mechanism standardizes processes of medical semantics. A repository that includes the definition of templates, archetypes, models, etc. can be found in the OpenEHR Clinical Knowledge Manager (CKM) [36].

## 3. Proposal of an IoMT Architecture and Platform to Enable Pervasive Healthcare

**Figure 3:**
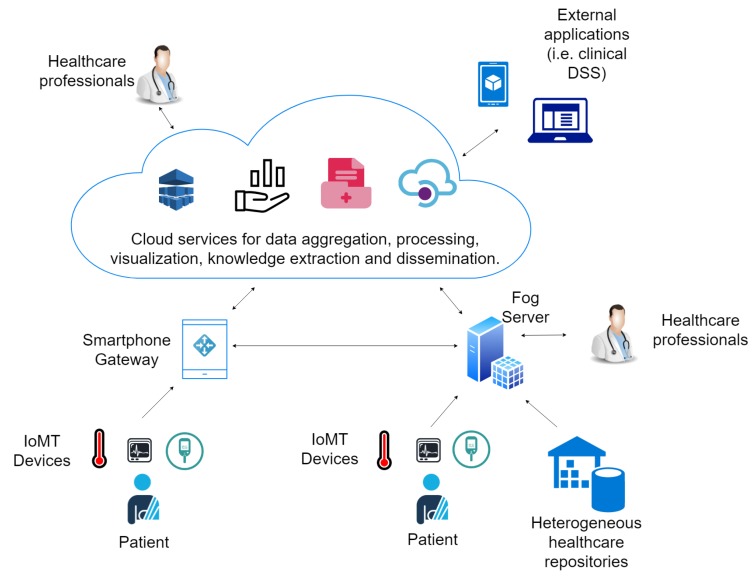
Typical IoMT-based e-Health system.

This section presents an IoMT architecture that considers the issues introduced above and describes the proposed IoMT platform. Figure 3 shows an overview of the main components of a cloud and an IoMT-based electronic health system followed for platform conception. Data are collected by heterogeneous sensors or extracted from healthcare repositories and sent to gateways or fog servers that act as a relay to the cloud. Patients’ smartphones are considered gateways for monitoring in patients’ environments. On the other hand, fog servers enable the instantiation of the system in hospitals or clinics playing the role of local hospital information systems (HIS) and enable the management and visualization of electronic records. The data are aggregated in the cloud, anonymized and processed for knowledge extraction and statistics analysis by tools based on big data processing, machine learning and online analytics processing. Moreover, sharing services are provided in the cloud for the dissemination of the data to third party applications.

### 3.1. Architecture Overview

**Figure 4:**
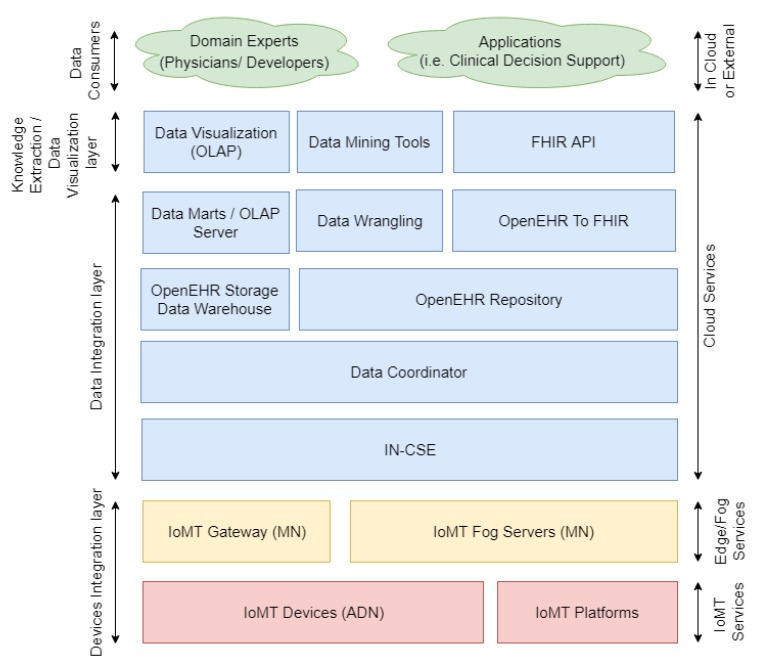
Platform Architecture.

The platform follows a three-layer architecture framed by the arrows on the left side of Figure 4 and defined as devices integration layer, data integration layer, and knowledge extraction and data visualization layer.

The data consumers are not part of the platform, since it represents external applications and consumers, such as physicians who use the platform. It has been included in Figure 4 for clarifying the way applications and external consumers can interact with the platform.

#### 3.1.1. Devices Integration Layer

This layer administers and integrates heterogeneous sensors and other clinical data sources (i.e., other EHRs or non-standardized HIS).

The following three different types of data sources were considered: (i) OneM2M compatible IoMT devices, which forward sensed data to the OneM2M gateway; (ii) non-OneM2M IoMT devices, which are independent and neither follow the OneM2M standard, nor belong to an IoMT platform; and (iii) an IoMT platform data source entity, which represents IoMT platforms that handle heterogeneous healthcare data (EHRs available in HIS of healthcare institutions or other healthcare repositories).

The last two types are integrated by data adapters that poll the data and transform them for OpenEHR. An adapter must be developed for each different data source and can be hosted either on the network edge (i.e., in an IoMT fog server/gateway), or in the cloud (following an infrastructure and platform as a service in the IN-CSE).

#### 3.1.2. Data Integration Layer

The data integration layer is responsible for the storage, maintenance, processing, and availability of the data. The CSE, inherited from OneM2M, provides a set of services for the collection of data gathered by OneM2M IoMT devices. In contrast, the Adapters enable the forwarding of the records gathered from the other data sources. Both interact with the data coordinator, which updates the OpenEHR repository and OpenEHR storage data warehouse. Then, (i) the data marts on the OLAP server update the data with the new record; (ii) the data wrangling entity prepares the data for the application of data mining tools; and (iii) the OpenEHR record is mapped to FHIR resources for the sharing of data through FHIR API.

All entities in this layer are instantiated in the cloud. However, the adapters can be instantiated on gateways and fog servers.

#### 3.1.3. Knowledge Extraction and Data Visualization Layer

This layer provides tools for the extraction of insights and visualization of data. The data visualization entity enables data inspection by providing visual interfaces that handle OLAP cubes. Therefore, physicians can apply analytic processing over the high volume of data gathered by multiple institutions and IoMT systems easily.

Data mining and machine learning tools enable the application of big data-processing techniques, such as Hadoop Map Reduce, and the training of several machine learning models, e.g., decision trees, neural networks, clusters, lineal, and non-lineal regressions, among others. This entity provides an API for the dissemination of previously trained models. Finally, FHIR API exposes the REST endpoints, which enables external applications to extract the EHRs standardized by the FHIR definition.

### 3.2. Description and Implementation of the Platform

**Figure 5:**
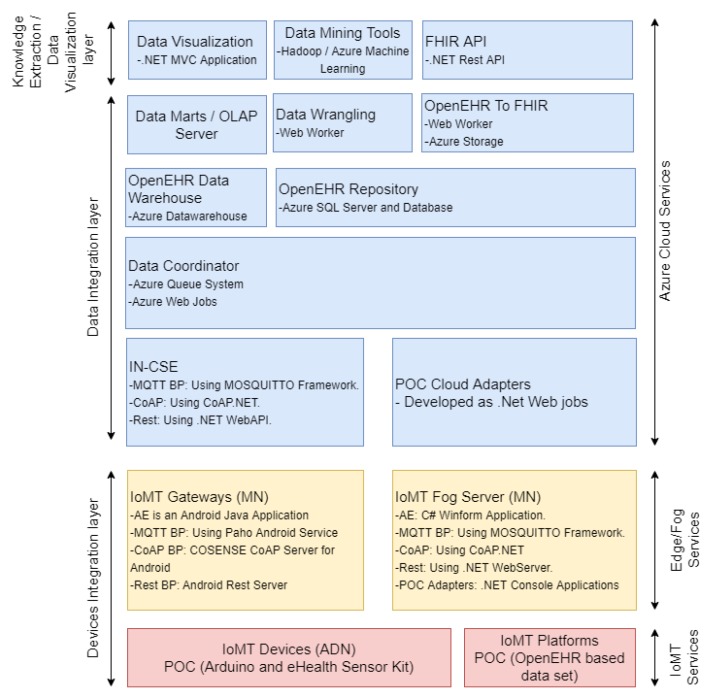
Technologies involved in platform implementation.

This section describes the main technical and functional characteristics of the platform. A summary of the main technologies involved and proof-of-concept development are shown in Figure 5 and detailed in the next subsections.

#### 3.2.1. IoMT Device (ADN)

**Figure 6:**
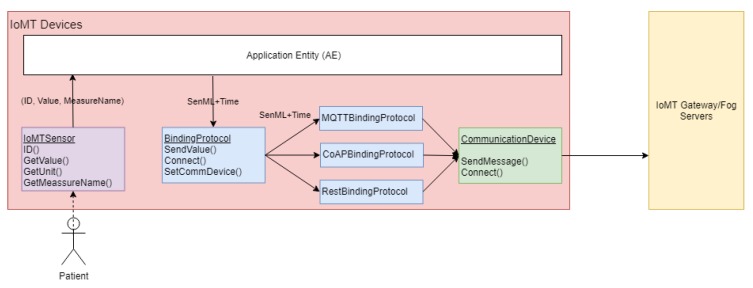
Development framework operation for an IoMT Device.

Since the IoMT devices follow the OneM2M specification, we provide a development framework written in an object-oriented paradigm in C++, which simplifies the development process of the AE and Binding Protocols (BPs). Figure 6 displays its main classes.

The IoMTSensor abstract class must be extended to represent the physical sensors and must implement functions to gather the sensor ID (obtained in the gateway and fog server after a registration process), read an observation value, retrieve the unit of measure associated with the observations, and obtain a friendly measure name (i.e., temperature, blood pressure, among other physical parameters’ names).

The BindingProtocol abstract class must be extended to represent novel or custom OneM2M binding protocols redefining the SendValue, Connect, and Set Communication Devices. The development framework already provides extensions of this class for MQTT, CoAP, and REST BPs. The communication must be implemented to have physical network access, and the framework enables implementations for Wi-Fi, GSM and Ethernet modules.

The ApplicationEntity class provides functionalities that connect the previous concepts and manages the monitoring process, such as conversion from sensor observation to SenML (Sensor Markup Language) data format and time tracking through the Universal Coordinated Time format and Network Time Protocol (NTP) services.

**Figure 7:**
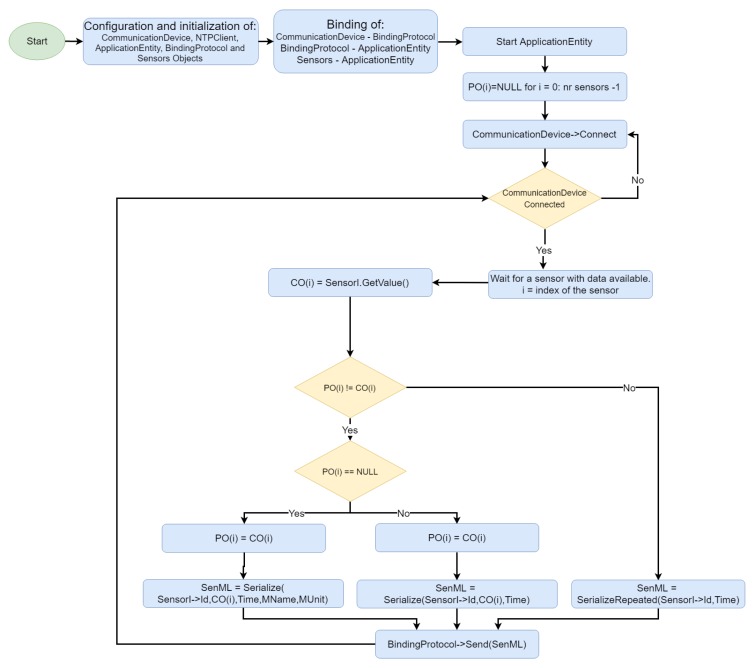
Initialization and message handling on IoMT devices using the development framework.

Figure 7 shows an example of the development framework operation for an IoMT device. Initially, the communication device, NTP client, AE, BP, and sensors are instantiated; the AE starts to run, and the communication device connects to the physical network. An IoMT device was designed as a platform that can host more than one sensor, each of them identified with an Id field. AE monitors the sensors until an observation CO(i) is available for the ith-sensor. CO(i), the current observation is then serialized as a SenML message and forwarded to either the gateway, or the fog server through the binding protocol.

SenML is serialized in three different ways to minimize the consumption of resources (mainly network overhead). An array PO[sensorcount] containing the previous observation made by each sensor and initialized with NULL values is maintained in the AE. If PO(i) is equal to a NULL value (i.e., first observation for sensor i), the SenML message will include fields for the sensor Id, current observation CO(i), time, friendly measure name (i.e., body temperature) and unit of measure. IfPO(i) has a value, SenML will contain the fields relative to sensor Id, observation O(t) and time, thus reducing the payload length. Moreover, if PO(i) is equal to CO(i), the observation does not need to be resent and the SenML message will contain only fields representing the sensor Id, time and a special flag that informs the gateway/fog server on CO(i) being equal to PO(i). The PO(i) value is updated with the CO(i) value whenever a message from a sensor *i* is sent.

**Figure 8:**
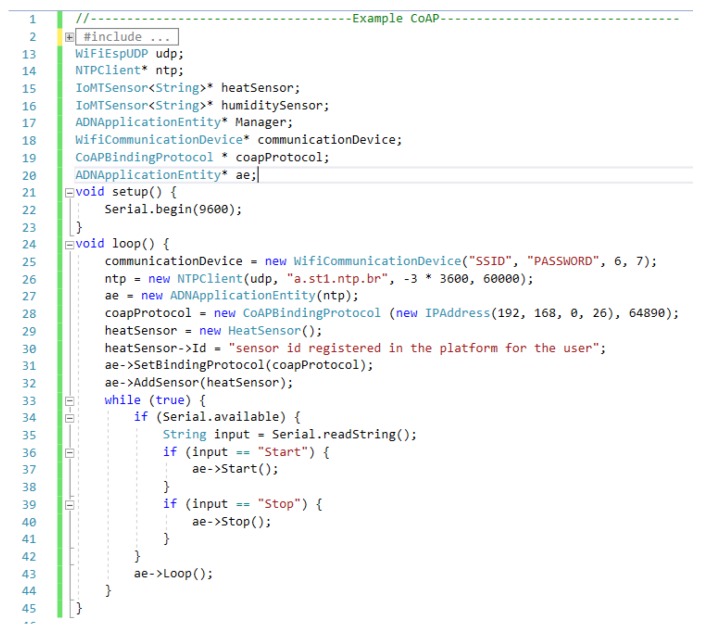
Example of coding of the development framework for the IoMT device.

Finally, Figure 8 shows an example code for an Arduino-based IoMT device that uses the CoAP BP provided in the development framework and a temperature sensor; in this case, less than 40 lines of code are necessary for integrating a device to our platform. The Id is configured in line 30 for identifying the sensor within the platform. In this example, it is defined via a code; however, it can be configured through a visual interface or another type of input inserting its value obtained after the registration of the patient and the sensors associated with it on the platform. The next section describes the process.

#### 3.2.2. IoMT Gateway (MN)

The development stage of the platform considered only gateways based on smartphones, widely used in IoT, M2M and IoMT scenarios ([37,38,39]).

Smartphones avoid the need for an additional equipment to be transported by the user and improve the platform in terms of support for users’ mobility. Moreover, end users can download the components from the different application stores as common applications.

The platform considered, initially, Android smartphones as gateways. According to [40], in July 2019, platforms based on the Android operating system dominate about 80% of the world market and the other 20% are distributed among Apple iOS, Microsoft Windows, and other lesser used operating systems. In addition to Android-based gateways, we are currently working on the implementation of the framework for Apple iOS and for an Arduino-based low-cost gateway.

IoMT gateways are considered OneM2M MNs, which host AEs, and CSEs. We provide a gateway development framework composed of an Android application skeleton that represents the AE and enables gateway configuration, user and sensor management, and data visualization. Gateways can operate as either a message relay between the ADN and the IN-CSE through the binding protocols available in the IN-CSE, or a bridge between ADNs and fog servers. The CSE is implicitly developed inside the AE and provides classes such as those proposed for the IoMT device, which enables the extension of binding protocols. Below are some of the steps required for a gateway configuration;

1. Supply of an IP address or URL of IN-CSE (cloud) or CSE (fog) (operation mode implicitly detected);
2. Registration on the platform for the supply of patient-related data, such as full name, birthday, gender, email, password, address, etc.;
3. Login to the platform by means of users’ credentials;
4. Registration of a sensor and obtaining of the sensor Id; and
5. Setting of the sensor Id to the IoMT device.

**Figure 9:**
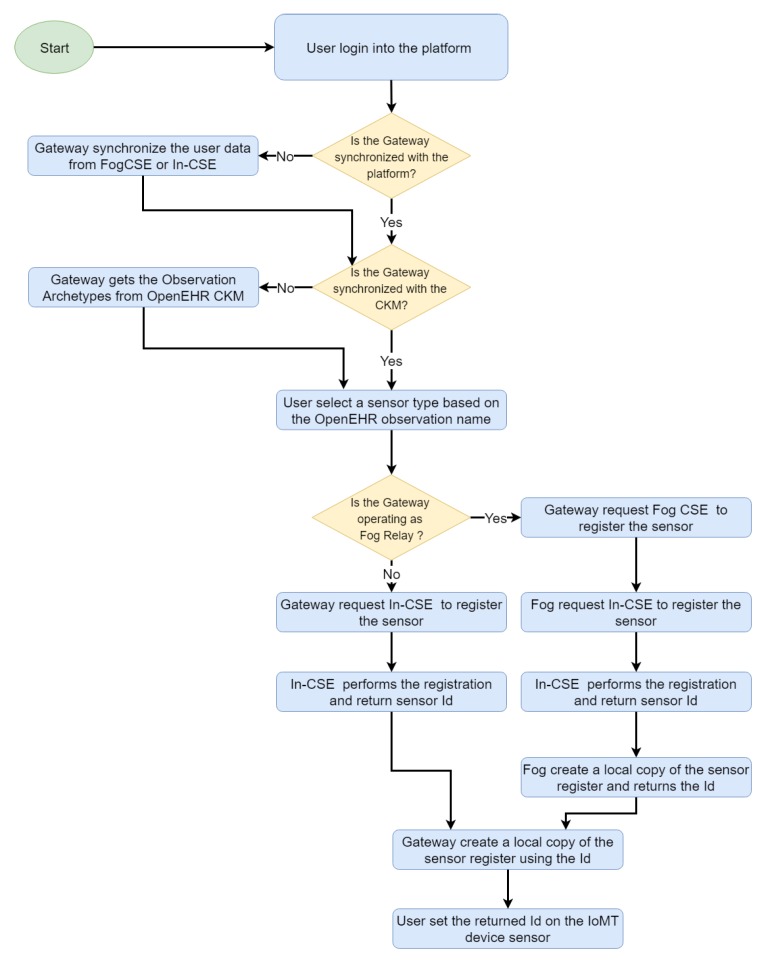
Sensor registration process (Gateway).

The sensor registration process demands particular attention. On our platform, sensor measures are associated with observations defined in OpenEHR, which enables the detection of invalid or damaged messages through an automatic process that validates an observation (and the sensor that generated it) that complies with all the restrictions defined in the observation archetype (detailed in Section 3.2.3 and Section 3.2.4). Figure 9 shows the sensor registration process, where the sensor Id is always generated by the cloud entity for avoiding persistence problems, and propagated to the fog, gateway and IoMT device. Each entity stores a record that maps the sensor Id to an OpenEHR archetype Id, which helps in the identification of type of data provided by the sensor and constraints to be fulfilled by the data.

**Figure 10:**
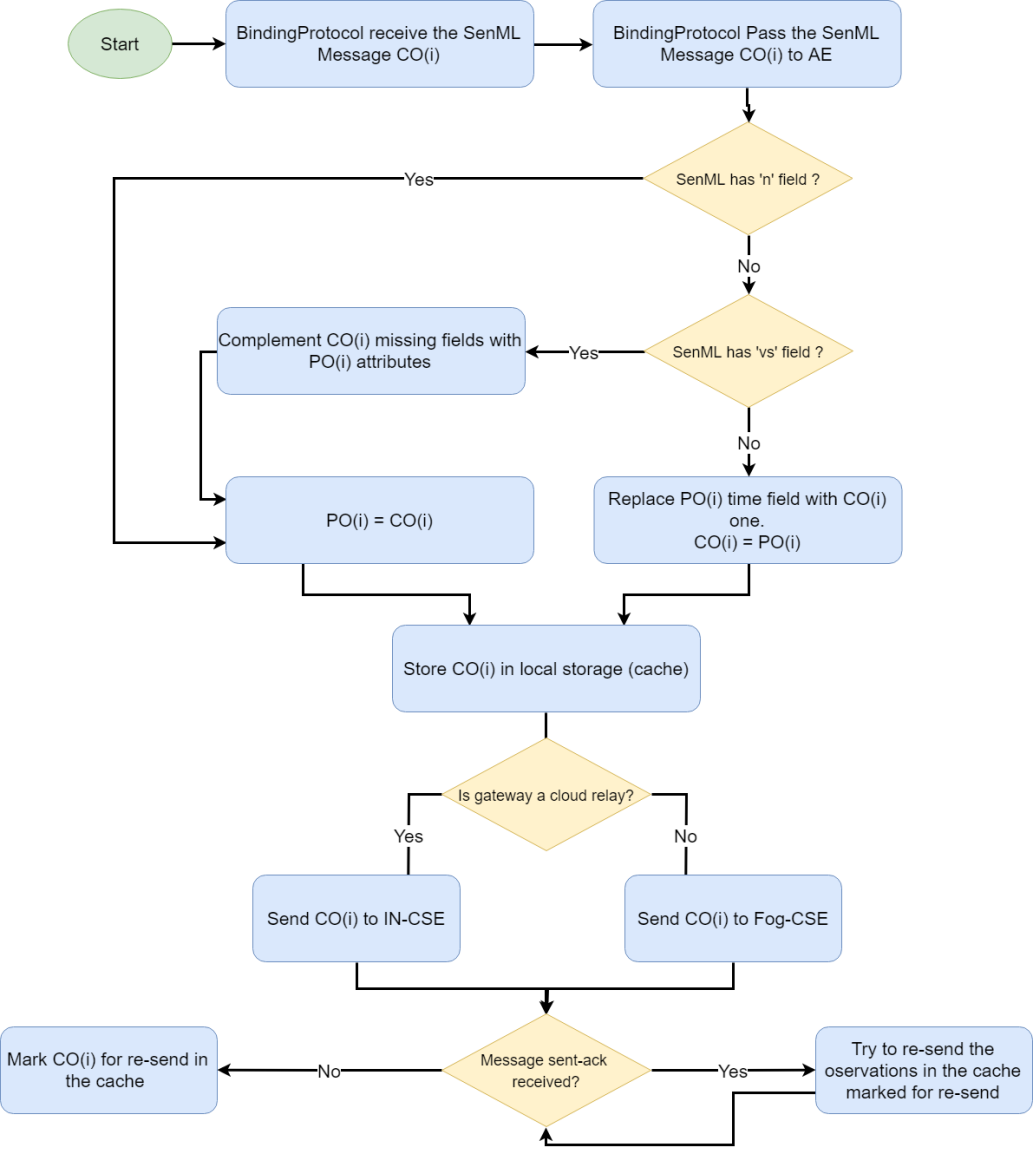
Message handling (Gateway).

Once the sensors have been registered and settled in the IoMT devices, the three types of SenML messages are handled (see Figure 10). The gateway stores data on the measure name and unit of measure provided by the first message for completing the subsequent compacted ones. Moreover, when the gateway detects a SenML message marked as repeated, it generates a new one containing all the fields, but using the current time. The gateway also provides other functionalities for data persistence, e.g., an offline local cache implemented to be used by the platform in scenarios of poor connectivity.

#### 3.2.3. IoMT Fog Server

The IoMT fog server was developed as a Microsoft .NET Web application (AE) and a set of REST endpoints (CSE) and acts similarly to the IoMT gateway. It was conceived as a common hospital information system to be deployed locally in a hospital or a clinic, and enables the management of users, IoMT devices, and navigation registration, edition, and deletion of EHR records. It can also operate as a master system independently of the cloud, or as a message relay between IoMT devices/gateways and the IN-CSE.

The fog server implements the same binding protocols present in the gateway, and other BPs can be deployed through the AE. Binding protocols are deployed as zipped files that contain executable applications with .exe, .py, .js and .bat extensions. The standard output of such applications, as well as the status of the BP, are shown on the AE which stops and starts them. Custom BPs interact with the platform via the CSE (post message endpoint) or through a file system queue. Messages posted in both approaches are treated as common messages received from IoMT devices.

Since the custom BPs are common applications, the platform can be extended with any type of system, since such BPs only need to implement a custom translator from the original format to SenML. Fields, such as device Id and user Id can be created and retrieved via the CSE.

**Figure 11:**
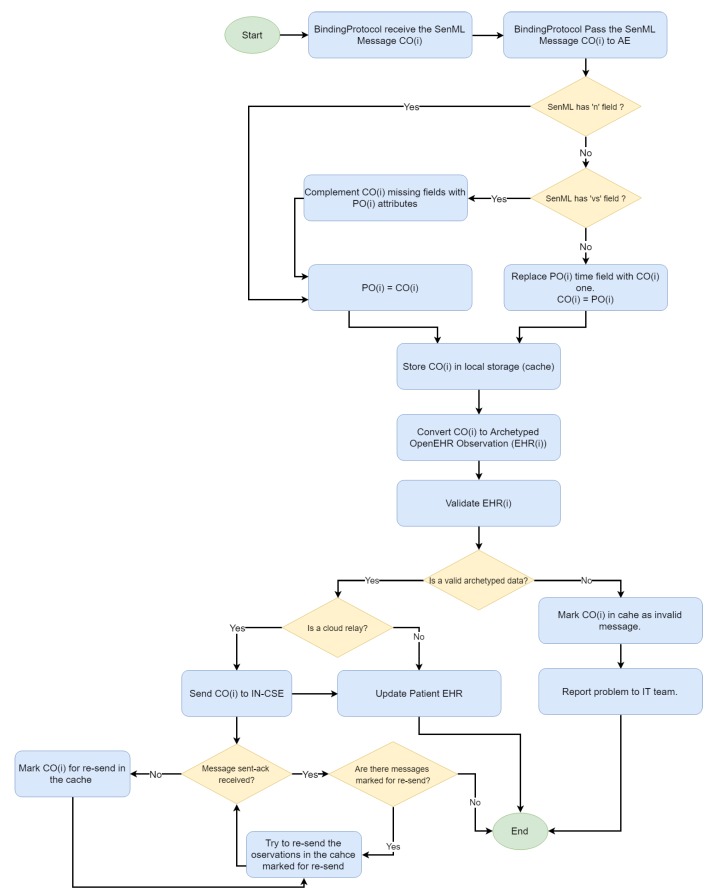
Message handling (Fog server).

The processing of the fog server messages follows an approach similar to the gateway one. According to Figure 11, the array of previous observations is maintained, and the handling of compacted and repeated messages is supported. However, the observations are converted to OpenEHR format and confronted with the OpenEHR definition for the checking of whether an observation complies with the restrictions defined in the semantic model. Only valid observations are stored and forwarded to the IN-CSE. A detailed report including the BP, device Id, violated constraint and other logs, is generated for troublesome observations; it enables developers to identify compatibility problems or those in the implementation of some of the custom BPs.

#### 3.2.4. Cloud Platform

**Figure 12:**
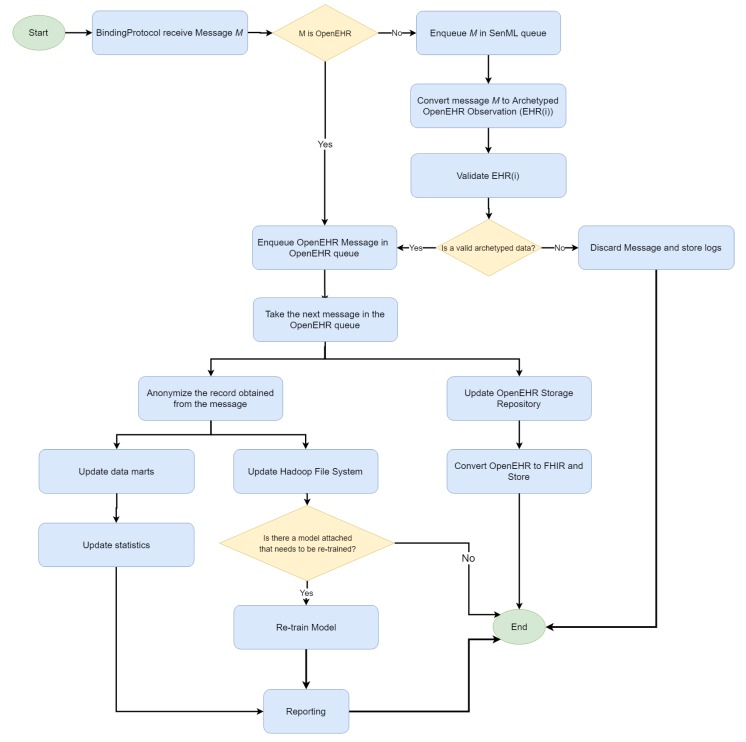
Message handling (Cloud services).

Finally, the cloud platform is provided as a service for a dynamic deployment of binding protocols (similarly to fog) and an adaptive infrastructure resources consumption. Two types of message can arrive in the IN-CSE. The first is the SenML encoded forwarded by the gateways, and the other refers to the OpenEHR records forwarded by the fog servers. Once a message has arrived in the IN-CSE, a process is triggered(see Figure 12).

Two queues were created for storing the messages, according to their SenML or OpenEHR format. SenML messages are dequeued and converted to OpenEHR ones, as in the fog server. A validation process is applied and, if the message is valid, it is moved to the OpenEHR queue. Such messages are used to populate a general OpenEHR storage composed of records from different hospitals or clinics that belong to the same institution. The OpenEHR records are also mapped to FHIR resources for an EHR interchange among institutions.

On the other hand, the OpenEHR archetyped data are anonymized and used to populate the data marts for online analytic processing and the Hadoop file system. Moreover, recurrent models attached to data mining tools (e.g., a recurrent neural network) receive the new record automatically. The platform enables the definition of dynamic data sets by composing data through the anonymized patient ID and/or OpenEHR observation types (identified by the archetype ID). The resulting data sets can be manually edited (e.g., for the classification of each record and training of a classification model) and used as input for the Machine Learning Accord.NET framework [41], which includes tools for classification, regression, clustering, serialization and sharing of trained models.

Finally, external applications can benefit from the pre-trained model. Moreover, healthcare professionals can visualize and analyze the synthesized data in a Data Visualization Web application that enables data filtering based on OpenEHR archetypes and extraction of several statistics.

### 3.3. Performance Evaluation

Several tests based on metrics, such as latency and resources (CPU and Memory) consumption were applied for validating the platform performance. The use case designed as Proof of Concept (POC) considers an IoMT device which combines a Cooking Hacks e-Health Sensor Kit development platform attached to an Arduino UNO and an ESP8266 Wi-Fi module to provide network access. The IoMT device comprises sensors, such as airflow, ECG, SpO2, electromyography, those related to blood pressure, blood sugar, body temperature, and an accelerometer that detects the patient’s position (sitting, lying, or standing).

**Figure 13:**
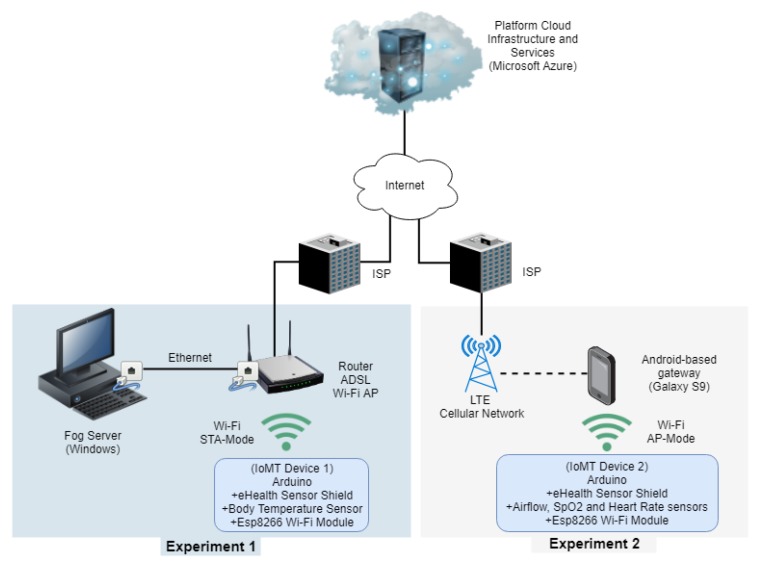
Network topology considered for test.

Figure 13 (Experiment 1) shows the network topology considered in the first test. The IoMT device connects through a Wi-Fi Access Point (Station Operation Mode) hosted in a router connected to the fog server via an Ethernet connection. The router provides ADSL Internet access and enables communication with the cloud services.

**Figure 14:**
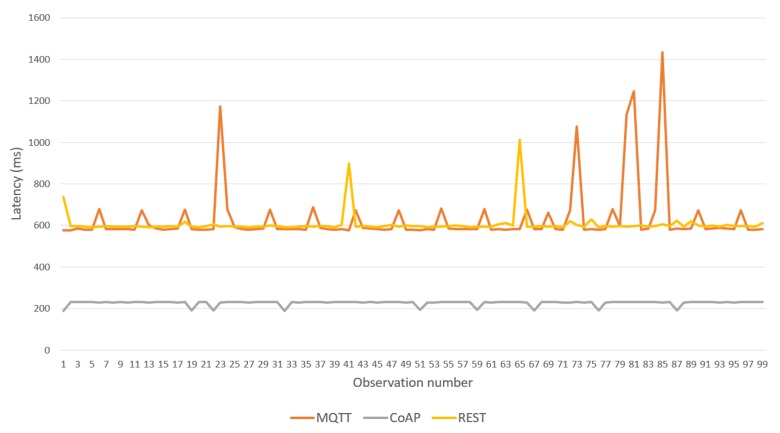
Latencies of the binding protocols over Wi-Fi (for IoMT devices<->Fog Server).

Over this topology, a sequence of 100 body temperature observations was transmitted from the IoMT device to the fog server, allowing comparison of the latency of the three BPs provided in the framework (see Figure 14). CoAP BP achieved the best performance and REST and MQTT BPs showed higher latency with similar results. Table 2 shows the maximum and average latencies for each BP. MQTT BP achieved the worst maximum latency; however, its average was close to that of the REST version. Table 3 shows the maximum RAM memory used in the IoMT device for each BP. Again, CoAP provided the best results. However, REST BP was better than MQTT.

The results motivate the use of CoAP BP and MQTT only if an external application is deployed on the fog server and consumes the data according to a publish/subscribe mode. REST BP should be considered only if the sensor has implemented a Web client and faster integration is required.

The second experiment considered the transmission latency between two IoMT devices and the IN-CSE on the cloud with a gateway acting as a relay. A device monitors the respiration rate defined in the OpenEHR archetype openEHR-EHR-OBSERVATION.respiration.v1, and the other monitors the saturation of oxygen in the peripheral blood, measured via pulse oximetry and the pulse/heart beat defined in archetypes openEHR-EHR-OBSERVATION.pulse-oximetry.v1 and openEHR-EHR-OBSERVATION.pulse.v1, respectively. As in the previous test, 100 observations of each parameter were collected and forwarded from the IoMT device to the cloud platform.

Figure 13 (Experiment 2) shows the network topology considered in this test. The IoMT device connects to a Samsung model S9 smartphone gateway through a Wi-Fi network (IoMT device operates as a Wi-Fi AP) and the gateway connects to the cloud platform through an LTE advance cellular network.

**Figure 15:**
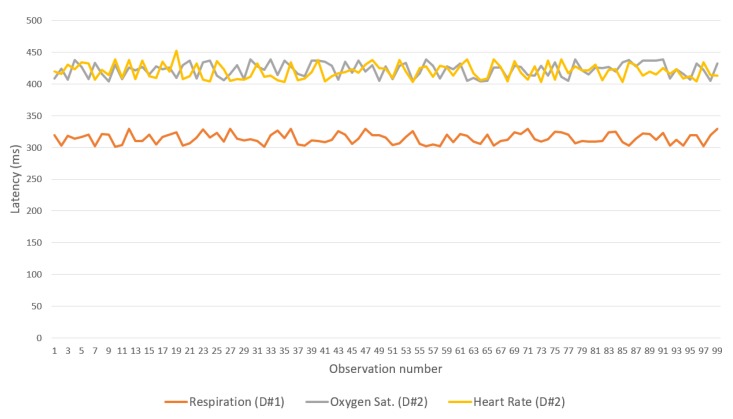
Latencies in the use of CoAP over Wi-Fi (IoMT devices<->Gateway).

**Figure 16:**
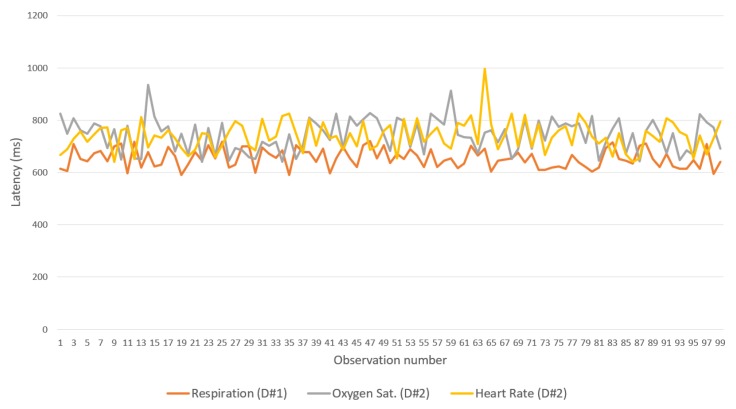
Latencies in the use of MQTT over Wi-Fi (IoMT devices-gateway).

**Figure 17:**
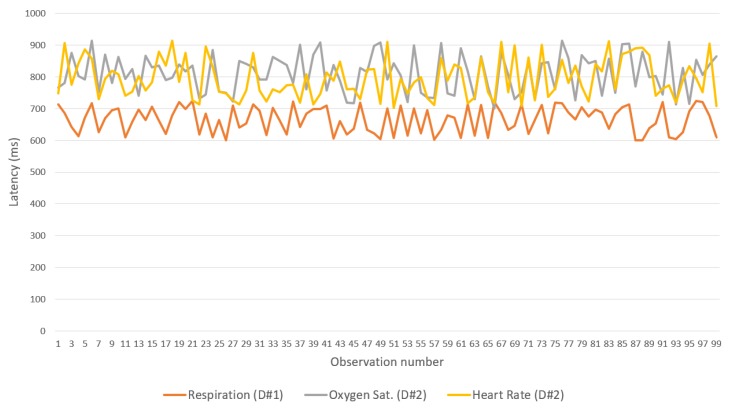
Latencies in the use of REST over Wi-Fi (IoMT devices<->gateway).

Figure 15, Figure 16 and Figure 17 show the latency between the IoMT devices and the gateway for each of the three flows and binding protocols. Again, CoAP achieved the best performance. A difference observed between the device that manages breathing data and the one that handles oxygen saturation and heart rate data is associated with the low processing resources available for the devices used—one of the devices has only one sensor, whereas the other has two.

**Figure 18:**
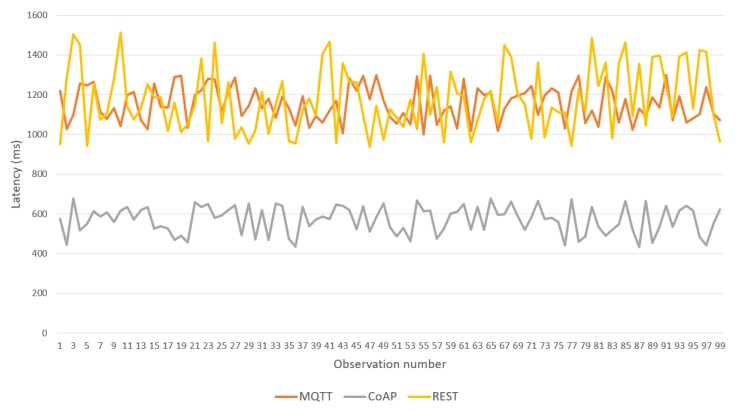
Latencies of binding protocols over LTE (for Gateway<->Cloud).

Finally, Figure 18 shows the latency between the gateway and the IN-CSE in the cloud. CoAP was considerably faster than the other two BPs. Therefore, the protocol is recommended for communication between gateways and IN-CSEs. However, all protocols showed good performance, with latencies lower than 1.5 s.

It is important to note that in the previous experiments the latency was measured in the application layer. It is influenced by the processing load on the IoMT device, the Fog Server and cloud platform. For example, the handling of SenML messages (Figure 10, Figure 11 and Figure 12) can add queuing and processing delays to the latency which was measured once the message had been completely processed. In addition, other processing loads related to the conversion of SenML messages into archetyped OpenEHR observations also have an impact on the results.

There are several studies ([42,43,44]) that validate the performance of the CoAP, MQTT and REST protocols considering the delivery of predefined payloads in an independent environment without interference from other actors and the results differ from those obtained in this manuscript. However, we prefer the validation of the proposed platform considering a real use scenario where factors such as those described above influence the performance. This allows the definition of better criteria regarding the performance of the platform.

A performance comparison with similar platforms would be interesting to better evaluate our proposal in terms of state-of-the-art. However, a fair comparison seems to be very difficult (or impracticable) given the differences in platform objectives, network architectures, and topologies, just to name a few features. Moreover, open source healthcare platforms were not identified. As such, a construction effort of other platforms would be needed, in order to make an effective comparison.

An additional issue was identified: many healthcare platforms focus their efforts on specific diseases, and several times use proprietary data formats and protocols, different from our proposal.

Our platform aims to cover the domain of healthcare as a whole, following widely adopted standards, enabling semantic interoperability as well as considering a big data approach.

Applications with ultra-low latency requirements may be affected considering the results. The better latencies were obtained using the CoAP BP which does not meet the requirements for ultra-low latencies applications, such as telesurgery or wireless service robots. In this sense the platforms enable the implementation of novel communications protocols as BPs, such as the proposed by [45]. Either way, the proposed platform is not focused on such types of applications and the experiments show encouraging results in terms of healthcare data standardization, processing, and sharing. For remote pervasive monitoring and wearable IoMT devices data aggregation, the platform meets the latency requirements (please see Table 1 in [46]).

## 4. Conclusions

IoMT is one of the novel approaches for enhancements in current healthcare services. Its use, combined with other paradigms, such as cloud computing, data mining, and machine learning, will empower physicians to make better decisions and improve knowledge of healthcare. However, several technical issues, addressed in this paper, hamper its application in the healthcare domain.

Our IoMT platform is based on an OneM2M architecture for e-Health applications. It enables the integration of multiple healthcare data sources and IoMT devices and sharing of healthcare knowledge according to the state-of-the-art tools for online analytic processing, big data, machine learning and healthcare data interchange.

Differently from other works, the proposed platform considers standardized data formats for the storage and transmission of data related to healthcare observations, which promotes interoperability regarding data representation formats, IoMT platforms and knowledge-extraction techniques.

In particular, OpenEHR helped the development of tools for the transformation of data in simple formats, such as SenML (almost raw data), into others, e.g., FHIR and OpenEHR itself. Since a sensor is attached to an OpenEHR observation definition, the correctness of an observation can be automatically evaluated according to a professional set of concepts. Moreover, a variation of the SenML format enabled the proposal of a transmission technique that avoids the sending of duplicated messages.

The platform automatically prepares the data for the application of OLAP through the anonymization of records, thus granting patient’s privacy. The integration of a Hadoop distributed file system simplifies the application of data mining, knowledge-extraction techniques and training of machine learning models based on pre-processed anonymized records resulting from the application of Map/Reduce algorithms over the healthcare repository.

A development framework was also implemented for the IoMT device, gateway, and fog server, for the integration of new sensors to the platform, extension of AE functionalities in the gateway, and implementation of new binding protocols. It enables the development of BP focused on data interchange with healthcare systems rather than isolated devices.

Finally, the performance tests yielded good results and validated the platform in the multiple scenarios considered. CoAP-based BP showed the lowest latency. Regarding MQTT and REST BPs, latency was shown as acceptable and considering the functional advantages of MQTT and the simplicity of REST, they can also be considered to be appropriate protocols.

Future studies will involve the analyses of other healthcare scenarios, such as Smart Cities (SC), where healthcare data play a vital role in the creation of statistical indicators of life quality and city performance. Moreover, analytical models for calculating throughput, mean time for data delivering and energy efficiency should be developed, as in [47,48].

## Figures and Tables

**Table 1:** Comparison of related works.

Reference	Objective	Enables APIs.	e-Health Standards	Interoperability	Big Data/Data Warehouse/OLAP Support
Boutros-Saikali et al. [21]	Heterogeneous healthcare repositories integration.	FHIR	FHIR Resource Format	Connectors	No
Cardoso et al. [24]	Dissemination and integration of information generated in hospital environments.	No	No	Agent	No
Kim [25]	Platform for ubiquitous healthcare.	Agent-based	ISO/IEEE 11073 Personal Health Data	Agent	No
Guan et al. [26]	Platform for link various ecosystems providing “interoperability as a service”.	REST API	No	Adapters	No
Irfan and Ahmad [27]	Review and proposal of an architectural model to implement Internet of Medical Things (IoMT).	No	No	Gateway Services/Fog and Edge Computing	No
Pramanik et al. [28]	Evaluation of various big data and smart system technologies in healthcare.	No	No	ETL over non-structured data	Big data management through Hadoop.
Essa et al. [29]	Identification and discussion on data-processing platforms that can be used in electronic health records	No	OpenEHR	ETL over EHR records	Data warehouse and OLAP cube
Essa et al. [29]	Identification and discussion on data-processing platforms that can be used in electronic health records	No	OpenEHR	Data wrangling over EHR records	Hadoop distributed file system and Map/Reduce

**Table 2:** Maximum and average latencies for each BP.

Binding Protocol	MQTT	CoAP	REST
Max. latency	1435 ms	233 ms	1014 ms
Avg. latency	629.80 ms	226.60 ms	606.16 ms

**Table 3:** Maximum RAM memory use of the IoMT device for each BP (kilobytes).

Binding Protocol	MQTT	CoAP	REST
Memory used	1696 kb	1138 kb	1451 kb
